# Serum caspase-3 levels and mortality are associated in patients with severe traumatic brain injury

**DOI:** 10.1186/s12883-015-0485-z

**Published:** 2015-11-06

**Authors:** Leonardo Lorente, María M. Martín, Mónica Argueso, Luis Ramos, Jordi Solé-Violán, Marta Riaño-Ruiz, Alejandro Jiménez, Juan M. Borreguero-León

**Affiliations:** Intensive Care Unit, Hospital Universitario de Canarias, Ofra, s/n. La Laguna, 38320 Santa Cruz de Tenerife, Spain; Intensive Care Unit, Hospital Universitario Nuestra Señora de Candelaria, Crta del Rosario s/n, Santa Cruz de Tenerife, 38010 Spain; Intensive Care Unit, Hospital Clínico Universitario de Valencia, Avda. Blasco Ibáñez n°17-19, Valencia, 46004 Spain; Intensive Care Unit, Hospital General de La Palma, Buenavista de Arriba s/n, Breña Alta, La Palma, 38713 Spain; Intensive Care Unit, Hospital Universitario Dr. Negrín, CIBERES, Barranco de la Ballena s/n, Las Palmas de Gran Canaria, 35010 Spain; Servicio de Bioquímica Clínica, Complejo Hospitalario Universitario Insular Materno-Infantil, Plaza Dr. Pasteur s/n, Las Palmas de Gran Canaria, 35016 Spain; Research Unit, Hospital Universitario de Canarias, Ofra, s/n. La Laguna, 38320 Santa Cruz de Tenerife, Spain; Laboratory Deparment, Hospital Universitario de Canarias, Ofra, s/n. La Laguna, 38320 Santa Cruz de Tenerife, Spain

**Keywords:** Caspase-3, Patients, Mortality, Injury, Brain

## Abstract

**Background:**

Different apoptosis pathways activate caspase-3. In a study involving 27 patients with traumatic brain injury (TBI), higher caspase-3 levels were found in contusion brain tissue resected from non-survivors than from survivors. The objective of this study was to determine whether there is an association in TBI patients between serum caspase-3 levels (thus using an easier, quicker, less expensive and less invasive procedure) and mortality, in a larger series of patients.

**Methods:**

We carried out a prospective, observational and multicenter study in six Spanish Hospital Intensive Care Units including 112 patients with severe TBI. All had Glasgow Coma Scale (GCS) scores lower than 9. Patients with an Injury Severity Score (ISS) in non-cranial aspects higher than 9 were excluded. Blood samples were collected on day 1 of TBI to measure serum caspas-3 levels. The endpoint was 30-day mortality.

**Results:**

We found that non-surviving patients (*n* = 31) showed higher (*p* = 0.003) serum caspase-3 levels compared to survivors (*n* = 81). Kaplan-Meier survival analysis showed a higher risk of death in TBI patients with serum caspase-3 levels >0.20 ng/mL than in patients with lower concentrations (Hazard Ratio = 3.15; 95 % CI = 1.40 to 7.08; *P <* 0.001). Multiple logistic regression analysis showed that serum caspase-3 levels > 0.20 ng/mL were associated with mortality at 30 days in TBI patients controlling for Marshall CT classification, age and GCS (Odds ratio = 7.99; 95 % CI = 2.116 to 36.744; *P =* 0.001).

**Conclusions:**

The association between serum caspase-3 levels and mortality in TBI patients was the major novel finding of our study.

## Background

Traumatic brain injury (TBI) is an important cause of death, disability, and health resource consumption [1]. Head trauma causes two types of injury in the neural tissue. One is the primary injury, which refers to the initial physical forces applied to the brain at the moment of impact. The other is the secondary injury, which develops over a period of hours or days later, involving neuroinflammatory response, free radical generation and apoptosis.

The apoptotic process is one in which cells are actively eliminated by a programmed pathway during morphogenesis, tissue remodeling, and resolution of the immune response; and it is increased in TBI [2–5]. Apoptotic cell death occurs mainly through two different pathways: the extrinsic or death receptor pathway (type I cells), and the intrinsic or mitochondrial pathway (type II cells). Both pathways, intrinsic and extrinsic, activate caspase-3 which leads to death cell.

Increased caspase-3 in brain tissues has been found in animal models of TBI [6–14]. However, caspase-3 has been scarcely explored in TBI patients, in small studies and only in brain tissue or cerebrospinal fluid (CSF) [15–19]. Higher caspase-3 levels in CSF have been found in TBI patients than in controls [15–17], and in brain tissue of TBI patients than in controls [18, 19]. In one study involving 27 TBI patients, higher caspase-3 levels were found in the brain tissue of non-surviving than in surviving TBI patients [19]. Thus the objective of this study was to determine whether there is an association between serum caspase-3 levels and mortality in TBI patients, (thus using an easier, quicker, less expensive and less invasive procedure compared with the determination in CSF or brain tissue of previous studies), in a larger series of patients.

## Methods

### Design and subjects

We carried out a prospective, observational and multicenter study in six Spanish Hospital Intensive Care Units including 112 patients with severe TBI. The study was approved by the Research Ethics Committees of the 6 participating hospitals, which were: Hospital Universitario de Canarias (La Laguna), Hospital Universitario Nuestra Señora de Candelaria (Santa Cruz de Tenerife), Hospital Clínico Universitario de Valencia (Valencia), Hospital General de La Palma (La Palma), Hospital Universitario Dr. Negrín (Las Palmas de Gran Canaria), and Hospital Insular (Las Palmas de Gran Canaria). Written informed consent was obtained from the patients or their legal guardians.

The present study included patients with severe TBI, defined as Glasgow Coma Scale (GCS) lower than 9 points [20]. We excluded patients with Injury Severity Score (ISS) [21] in non-cranial aspects higher than 9 points, age less than 18 years, pregnancy, inflammatory or malignant disease.

### Variables recorded

The following variables were recorded for each patient: brain lesion according to Marshall computed tomography (CT) classification [22], sex, age, Acute Physiology and Chronic Health Evaluation II (APACHE II) score [23], activated partial thromboplastin time (aPTT), bilirubin, cerebral perfusion pressure (CPP), creatinine, fibrinogen, GCS, glycemia, hemoglobin, ICP, international normalized ratio (INR), ISS, lactic acid, leukocytes, pressure of arterial oxygen (PaO2), fraction of inspired oxygen (FI0_2_), platelets, sodium, and temperature. The endpoint of the study was 30-day mortality.

### Determinations of caspase-3 serum levels

Blood samples were collected on day 1 of TBI (within the first 4 h after TBI) in tubes with separator gel. After coagulation during 10 min at room temperature, serum was obtained by centrifugation at 1000 g for 15 min. The samples were aliquoted and frozen at −80 °C until determination. All determinations were performed by laboratory technicians blinded to all clinical data. Assays were performed at the Laboratory Department of the Hospital Universitario de Canarias (La Laguna, Tenerife, Spain).

Caspase-3 levels were measured in serum by solid-phase enzyme-linked immunosorbent assay (ELISA) quantitative sandwich using Human Caspase-3 Elisa BlueGene Biotech® (Shanghai, China). The intra- and inter-assay coefficients of variation (CV) were <5.6 % and <7.9 % respectively. The detection limit for the assay was 0.1 ng/mL.

### Statistical methods

Quantitative variables are reported as medians and interquartile ranges, and were compared with Wilcoxon-Mann–Whitney test. Qualitative variables are reported as frequencies and percentages and compared using Chi-squared test.

We used receiver operating characteristic analysis to determine the goodness-of-fit of serum caspase-3 levels to predict 30-day mortality. We carried out a Kaplan-Meier analysis to compare 30-day survival according to serum caspase-3 levels lower/higher than 0.20 ng/mL.

We constructed predictive Cox regression models of survival after TBI. The variables included were: serum caspase-3 levels > 0.20 ng/mL, Marshall CT classification, GCS and age as independent variables. To include the variable CT classification in the regression analysis, it was recoded according to the risk of death observed in the bivariate analysis as high risk of death (CT types 3, 4 and 6) and low risk of death (CT types 2 and 5). The dependent variable was time to death. We included −2 log likelihood ratios to compare the models. We used Hazard ratio and 95 % confidence interval (CI) to estimate the clinical impact of the predictor variables.

SPSS 17.0 (SPSS Inc., Chicago, IL, USA) and LogXact 4.1, (Cytel Co., Cambridge, MA) was used to perform statistical analyses. All *P* values lower 0.05 were considered statistically significant

## Results

At 30 days after TBI, 31 of the 112 (27.7 %) patients had died. We observed a lower proportion of female patients (*p* = 0.02) among surviving than non-surviving TBI patients, and different CT findings (*p* = 0.003) (Table 1). Surviving TBI patients showed higher GCS (*p* < 0.001), lower age (*p* < 0.001), and lower APACHE-II score (*p* < 0.001) than non-survivors. In addition, surviving patients showed lower serum caspase-3 levels than non-survivors (*p* = 0.003) (Table 2).

**Table 1:** Comparison of computed tomography findings and gender between surviving and non-surviving patients with traumatic brain injury

	Survivors (*n* = 81)	Non-survivors (*n* = 31)	*P*-value
Marshall CT classification - n (%)			0.003
Type 6	5 (6.2)	8 (25.8)	
Type 5	26 (32.1)	5 (16.1)	
Type 4	12 (14.8)	9 (29.0)	
Type 3	14 (17.3)	6 (19.4)	
Type 2	24 (29.6)	3 (9.7)	
Type 1	0	0	
CT findings with high risk of death (types 3,4,6) - n (%) with classification - n (%)	31 (38.3)	23 (74.2)	0.001
Female gender – n (%)	13 (16.0)	12 (38.7)	0.02

Data are shown as number and percentage; *CT* Computed tomography

**Table 2:** Comparison of clinical and biochemical characteristics between surviving and non-surviving patients with traumatic brain injury

	Survivors (*n* = 81)	Non-survivors (*n* = 31)	*P*-value
Age (years)	46 (27–60)	63 (53–75)	<0.001
APACHE-II score	18 (15–22)	26 (24–29)	<0.001
aPTT (seconds)	28 (25–31)	29 (25–36)	0.86
Bilirubin (mg/dl)	0.50 (0.40–0.80)	0.70 (0.58–0.95)	0.045
Caspase-3 (ng/ml)	0.11 (0.10–0.18)	0.23 (0.10–0.46)	0.003
CPP (mmHg)	68 (57–70)	61 (54–69)	0.48
Creatinine (mg/dl)	0.80 (0.63–1.00)	0.80 (0.70–1.10)	0.44
Fibrinogen (mg/dl)	366 (283–448)	360 (269–520)	0.32
Glasgow Coma Scale score	7 (5–8)	3 (3–6)	<0.00171
Glycemia (g/dL)	139 (122–167)	160 (134–189)	0.08
Hemoglobin (g/dL)	11.4 (10.2–13.0)	11.9 (9.8–13.1)	0.87
ICP (mmHg)	15 (14–20)	25 (13–34)	0.28
INR	1.11 (1.00–1.21)	1.12 (1.03–1.40)	0.29
ISS	25 (25–29)	25 (25–29)	0.80
Lactic acid (mmol/L)	1.70 (1.10–2.50)	2.40 (1.30–4.60)	0.06
Leukocytes *10^3^/mm^3^	14.5 (10.3–19.0)	16.3 (9.8–22.7)	0.46
PaO2 (mmHg)	148 (110–203)	133 (98–180)	0.33
PaO2/FI0_2_ ratio	336 (240–400)	274 (173–393)	0.11
Platelets *10^3^/mm^3^	184 (134–244)	180 (125–237)	0.52
Sodium (mEq/L)	140 (138–143)	142 (138–148)	0.19
Temperature (°C)	37.0 (36.0–37.3)	36.0 (35.0–37.0)	0.12

Data are shown as median (percentile 25^th^–75^th)^; *APACHE II* Acute Physiology and Chronic Health Evaluation, *aPTT* activated partial thromboplastin time, *CPP* cerebral perfusion pressure, *ICP* intracranial pressure, *INR* international normalized ratio, *ISS* Injury Severity Score, *PaO*_*2*_ pressure of arterial oxygen, *FIO*_*2*_ fraction of inspired oxygen

On receiver operating characteristic analysis, the area under the curve of serum caspase-3 levels to predict morality at 30 days in TBI patients was 0.68 (95 % CI = 0.56–0.80; *P =* 0.003).

**Fig. 1:**
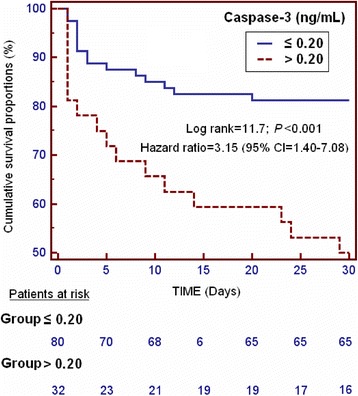
Survival curves at 30 days using serum caspase-3 levels higher or lower than 0.20 ng/mL

Kaplan-Meier survival analysis showed a higher risk of death in TBI patients with serum caspase-3 levels >0.20 ng/mL than in patients with lower concentrations (Hazard Ratio = 3.15; 95 % CI = 1.40 to 7.08; *P <* 0.001) (Fig. 1).

Cox regression analysis showed that serum caspase-3 levels >0.20 ng/mL were associated with mortality at 30 days in TBI patients after controlling for Marshall CT classification, age and GCS (Hazard ratio = 2.956; 95 % CI = 1.365 to 6.403; *P* = 0.006) (Table 3).

**Table 3:** Cox regression analysis to predict mortality

	−2 log likelihood ratio	Hazard Ratio	95 % Confidence Interval	*P*-value
Model 1:	274.431	3.163	1.562–6.405	0.001
Serum caspase-3 > 0.20 ng/mL				
Model 2:	263.541			
Serum caspase-3 > 0.20 ng/mL		2.916	1.438–5.917	0.003
CT findings (high vs low risk of death)		3.538	1.577–7.940	0.002
Model 3:	234.391			
Serum caspase-3 > 0.20 ng/mL		2.943	1.396–6.203	0.005
Glasgow Coma Scale		0.703	0.586–0.845	<0.001
Model 4:	240.152			
Serum caspase-3 > 0.20 ng/mL		3.349	1.605–6.990	0.001
Age (years)		1.039	1.017–1.061	0.001
Model 5:	209.615			
Serum caspase-3 > 0.20 ng/mL		2.956	1.365–6.403	0.006
CT findings (high vs low risk of death)		4.289	1.836–10.016	0.001
Glasgow Coma Scale		0.694	0.567–0.849	<0.001
Age (years)		1.036	1.015–1.058	0.001

*CT* Computed tomography

## Discussion

The novel findings of our study were that non-surviving TBI patients showed higher serum caspase-3 levels than survivors, that high serum caspase-3 levels were associated with higher mortality, and that serum caspase-3 levels could be used as a biomarker to predict mortality in TBI patients.

Previously, higher caspase-3 levels were found in the CSF of TBI patients than in controls [15–17], and in brain tissue of TBI patients than in controls [18, 19]. In our study we found higher serum caspase-3 levels in non-surviving than in surviving TBI patients. These results are in consonance with those of a study by Nathoo et al. [19] involving 27 TBI patients, which showed higher caspase-3 levels in the brain tissue of non-survivors than survivors. The new aspect of our study was that caspase-3 levels were measured for the first time in serum; thus using an easier, quicker, less expensive and less invasive procedure compared with the determination in CSF or brain tissue of previous studies. The major novel finding of our study, according to the results of Cox regression analysis, was that serum caspase-3 levels >0.20 ng/mL were associated with 30-day mortality in TBI patients.

The findings of our study suggest that apoptosis in patients with severe TBI may play an important role in prognosis. Apoptotic cell death occurs primarily through three different pathways: the extrinsic or death receptor pathway (type I cells), the intrinsic or mitochondrial pathway (type II cells), and the endoplasmic reticulum pathway [2–5]). In type I cells, the activation of a surface death receptor of tumor necrosis factor receptor superfamily (TNFRSF) by its cognate death ligand (TNFSF) initiates apoptosis, then a death signal is created and cleaves pro-caspase-8 in active caspase-8, which activates caspase-3. In type II cells, apoptosis could be initiated by cytokines such as interleukin (IL)-1 and IL-6, and oxygen free radicals; in this pathway the mitochondria release cytochrome c which activates caspase 3. Both apoptotic pathways, intrinsic and extrinsic, activate caspase-3 and that leads to cell death. Caspase-3 cleaved DNA fragmentation factor subunit alpha (DFFA), also known as Inhibitor of caspase-activated DNase (ICAD), and triggers DNA fragmentation during apoptosis [24].

In rat models, the administration of caspase-3 inhibitors have reduced caspase-3 activity and apoptosis in brain tissues [9–14]. Thus, from a therapeutic perspective, the use of modulators of apoptotic activity by inhibition of caspase-3 activity could be new class of drugs for the treatment of TBI.

Our study has certain limitations. First, the determination of circulating caspase-3 levels during follow-up was not performed and could be interesting. Second, we did not determine serum caspase-3 levels in healthy controls; however, the objective of our study was not to determine whether there is an increase of serum caspase-3 levels in TBI patients, but rather whether there is an association between serum caspase-3 levels and 30-day mortality in TBI patients. Third, the inclusion and exclusion criteria of our study were fairly restrictive, which hinders extrapolation of the findings to the general TBI population. However, we excluded patients with ISS in non-cranial aspects higher than 9 or inflammatory disease to avoid the influence of inflammation on serum caspase-3 levels; and we excluded patients with malignant disease to avoid the influence of therapeutic effort limitation on prognosis. Four, blood samples were collected on the day of TBI (within the first 4 h after TBI); however, the exact moment of blood sampling was not reported and the time between the moment of trauma and blood sampling may be different among patients. Five, the findings of our study suggest that there is an association between serum caspase-3 levels and mortality in TBI patients and that serum caspase-3 levels could be used as a prognostic biomarker of early mortality; however, these findings do not imply a causal relationship. Thus, additional studies are needed to confirm the results of our study

## Conclusions

The association between serum caspase-3 levels and 30-day mortality in TBI patients was the major novel finding of our study.

## Abbreviations

TBI: Traumatic brain injury
GCS: Glasgow Coma Scale
ISS: Injury Severity Score
PaO_2_: Pressure of arterial oxygen/fraction inspired oxygen
FIO_2_: Pressure of arterial oxygen/fraction inspired oxygen
aPTT: activated partial thromboplastin time
APACHE II: Acute Physiology and Chronic Health Evaluation
